# Robot deployment in long-term care: Case study on using a mobile robot to support physiotherapy

**DOI:** 10.1007/s00391-016-1065-6

**Published:** 2016-06-03

**Authors:** K. Gerling, D. Hebesberger, C. Dondrup, T. Körtner, M. Hanheide

**Affiliations:** School of Computer Science, University of Lincoln, Brayford Pool, LN6 7TS Lincoln, UK; Akademie für Altersforschung am Haus der Barmherzigkeit, 1160, Seeböckgasse 30a, Vienna, Austria

**Keywords:** Older adults, Assisted living facilities, Assistive technology, Robotics, Senioren, Hilfen im betreuten Wohnen, Assistive Technologie, Mensch-Roboter-Interaction

## Abstract

**Background:**

Healthcare systems in industrialized countries face the challenge of providing care for a growing number of elderly people. Information technology has the possibility of facilitating this process by providing support for nursing staff and improving the well-being of the elderly through a variety of support systems.

**Objective:**

Little is known about the challenges that arise from the deployment of technology in care settings; however, the integration of technology into care is one of the core determinants of successful support. This article presents the challenges and options associated with the integration of technology into care using the example of a mobile robot to support physiotherapy for elderly people with cognitive impairment in the European project Spatio-Temporal Representations and Activities for Cognitive Control in Long-Term Scenarios (STRANDS).

**Results and discussion:**

This article presents the technical challenges associated with the introduction of robots in the context of care as well as the perspectives of physiotherapists involved and an overview of information and experiences gained. It is hoped that this will provide useful information for the work of researchers and practitioners wishing to integrate robotic aids into the caregiving process.

## Zusammenfassung

**Hintergrund:**

Viele Gesundheitssysteme stehen vor der Herausforderung Pflegeleistungen für die steigende Zahl an alten Menschen zu gewährleisten. Informationstechnologien stellen eine vielversprechende Möglichkeit dar, diesen Anforderungen gerecht zu werden. Hierzu werden sie in verschiedenen Anwendungsformen zur Unterstützung von Pflegepersonal oder zur Steigerung des Wohlbefindens alter Menschen eingesetzt.

**Ziel:**

Bislang ist noch wenig über jene Herausforderungen bekannt, die sich im Zuge des Einsatzes von Technologien im Pflegebereich ergeben. Allerdings stellt aber gerade die Integration solcher Technologien im Pflegebereich einen der bestimmenden Faktoren hinsichtlich der künftigen Unterstützung des Pflegepersonals dar. In diesem Beitrag diskutieren wir daher Herausforderungen und Möglichkeiten, die mit der Integration von Technologien im Pflegekontext einhergehen. Dies geschieht anhand eines Fallbeispiels, in dem ein mobiler Roboter im Rahmen des EU-Projektes Spatio-Temporal Representations and Activities for Cognitive Control in Long-Term Scenarios (STRANDS) als Assistent im Bereich der Physiotherapie von alten Menschen mit fortgeschrittener Demenz eingesetzt wurde.

**Resultate und Diskussion:**

In diesem Paper wollen wir v. a. technische Herausforderungen, die mit dem Einsatz eines Roboters im Pflegekontext verbunden sind, sowie Perspektiven der involvierten Physiotherapeuten fokussieren, um einen Überblick über Erkenntnisse und Erfahrungen zu bieten. Damit hoffen wir, Wissenschaftlern und Praktikern wertvolle Informationen zu bieten, die im Zuge der Integration von assistiven Robotern im Pflegebereich künftig nutzbar gemacht werden können.

## Introduction to the topic

Assistive technology, including robots, has the potential to support caregiving for older adults in various ways, for example, by supporting nursing staff or providing mental and physical stimulation for residents of care facilities; however, it is important to consider the needs of care facilities, the residents and the extent to which currently available technology is able to provide support to develop accessible and acceptable systems. To this end it is important to consider challenges and opportunities that arise from the caregiving context and that result from technical limitations.

## Introduction

The growing number of older people in industrialized countries increases pressure on healthcare systems to provide care that is both affordable and meets the emotional and physical needs of older persons. In this context the integration of information technology holds promise of supporting caregiving processes: for example, assistive technology has been developed to provide reminders and empower individuals to live independently in their own homes [20], to encourage older adults to participate in preventative therapy [28] or software systems that can support staff in care facilities [29]. Findings from case studies (e. g. [6] and [29]) suggest that information technology is suited to support informal carers and professional care provides alike who are looking to provide quality care to aging populations; however, an important aspect that needs to be considered particularly when discussing the potential of information technology to support long-term care, are challenges that arise from the practical deployment of new technologies in caregiving environments. While assistive technology for older adults has made big advancements in the last few years, for example, the development of companion robots [2] and more comprehensive, intelligent systems to support home care (e. g. ambient-assisted living systems utilizing voice interaction as proposed by Portet et al. [23], there are a number of unique challenges that need to be considered when taking technology out of the laboratory environment into the field. To this end, this article provides insights from a case study around robotic support for older adults in long-term care that investigated the potential of a mobile robot to support physiotherapy in the large-scale European project Spatio-Temporal Representations and Activities for Cognitive Control in Long-Term Scenarios (STRANDS, see Appendix, section 1). Specifically, the case study explored whether older adults could be engaged in a Nordic walking group led by a robotic pacesetter who would guide them on indoor walks (see Appendix, section 2). This article aims to provide an overview of the practical and technical challenges on the basis of findings from the case study. We combine perspectives of care and technology experts to provide a comprehensive overview of relevant considerations and to discuss lessons learned with a focus on technical limitations, opportunities and practical challenges that need to be considered when integrating robots in long-term care. Thereby, we hope to provide valuable information that can be helpful for practitioners and researchers alike considering the benefits of technology integration in long-term care.

## Background

This section summarizes previous efforts in the development of technology to support well-being among older adults and discusses related work that has explored the deployment of technology in long-term care.

### Designing technology to support well-being of older adults

Throughout the last decades numerous attempts have been made to develop technology that contributes to the well-being of older adults, particularly focusing on supporting older persons while attending to individual personal needs and assistive technology to support independent living. For example, the potential of entertainment software and social networking tools was explored to support engaging leisure activities and to connect older persons with peers (e. g. [1]). In this context developers tried to integrate aspects of entertainment and socializing into preventative applications: for example, the study by Ogonowski et al. [21] investigated the potential of social, game-based tools to promote balance training and prevention of falls among older adults. Additionally, numerous research projects have looked into the development of ambient-assisted living solutions and other tools that can help older adults maintain independence. Among others, Grönvall and Verdezoto [9] explored tools to enable older adults to monitor blood pressure in their own homes and Lee and Dey [16] discussed systems designed to offer adults reminders to take medication. With respect to robotic deployment in the home, many studies focused on the requirements of older adults for robot deployment in domestic areas (e. g. [19]). The main findings indicate that older adults prefer robot assistance for instrumental tasks, such as housekeeping (e.g. garden work and cleaning), for manipulation of objects (e.g. picking up and moving heavy items, finding and fetching items) and reminder functions but preferred human assistance in terms of cooking, personal hygiene and leisure activities. In this context, Prakash et al. [24] explored the potential of robotic aids in the provision of medication. The findings suggest that older adults’ acceptance of the approach largely depends on their attitudes towards robots, outlining the variety of factors that play a role in the successful integration of robotic assistance.

While many of these results are promising in terms of the benefits that technology could have for older adults, further considerations regarding the suitability of such technologies for older adults are necessary to address challenges that arise from the practical deployment of technology in the homes of older adults and in residential care.

### Technology deployment in the home and in long-term care

A growing body of research addresses the development of technologies designed to be embedded in long-term care and there are numerous examples of technology deployment in caregiving environments, each associated with unique challenges. For example, Gerling et al. [7] studied the integration of console games as social activity in long-term care over the course of 4 months. The results of the project revealed that inviting residents to engage with technology in a social setting was problematic (e. g. participants felt self-conscious), suggesting that factors emerging from technology deployment in the environment of the specific care facility and abilities of residents need to be considered in addition to general aspects relating to the accessibility of technology for older adults. Addressing the needs of staff rather than directly focusing on those of older adults, Webster and Hanson [29] investigated the benefits of a software support system for staff in long-term care, suggesting that it could be of substantial assistance when interacting with residents by providing individual background information on resident needs for members of staff.

With respect to robotic deployment in long-term care, projects have previously explored the potential of robotic assistance, for example focusing on older adults living in retirement communities [22] or as a conversational aid in a care centre attended by older adults on some days of the week [26]. Focusing on ambient-assisted living solutions Caine et al. [3] investigated how older adults responded to the presence of monitoring technology, including a mobile robot and how it affected privacy enhancing behavior of older adults. The findings demonstrated that older adults adapted their behavior in the presence of such technologies in a way that allowed them to protect their privacy, suggesting that further research is necessary to facilitate seamless integration of technology into care and improving the acceptability and understanding of technology to the point where older adults do not feel like the need to modify their own behavior.

Finally, research has also begun to address needs of staff in long-term care facilities catering for older adults who experience substantial age-related changes and impairments. Work by Smarr et al. [27] presented a social assistive robot with entertainment and communication support functions for older adults and staff members. Results showed that staff perceived both tasks positively. In another study [19] older adults and care staff had to rate their preferences in a list of different predefined tasks. Findings showed that care staff prioritized tasks, such as lifting heavy objects, monitoring the location of people, switching electrical appliances or lights on or off, reminding of daily routines, escorting residents to meals or using the robot as a walking assistance for older adults; however, little is known about the practical requirements that would arise through robotic deployment. This shows that despite great advances in the general development of technology to support well-being among older adults, researchers and practitioners still face substantial challenges when integrating technologies into long-term care. In the remainder of this article we discuss an exemplary case study of robotic pacesetting for an indoor walking group. Thereby, we provide an overview of practical and technical challenges that need to be considered when integrating a robotic support system in a long-term care facility catering for older adults with cognitive impairment.

## Challenges and opportunities of robot deployment in long-term care

Within the 4‑year project STRANDS (see Appendix, section 1) an assistive robot was introduced into the long-term care hospital “Haus der Barmherzigkeit” in Vienna Austria. This institution primarily houses older adults with cognitive impairment and dementia (see Appendix, section 3). One aim of this project is to investigate methods of long-term adaptation and learning for mobile robots in the care context and other real world work environments and to study the social impact of the introduction of robot technologies to these areas of deployment. In this section, we provide an overview of challenges and opportunities that were identified in a collaborative process between researchers and staff from different professions throughout different deployment phases. In this context, we focus on results addressing caregiving-related aspects along with considerations that focus on limitations and the potential of robotic technology.

### Background requirements analysis and walking group deployment with robotic pacesetting

If robotic aids should support the care sector in future, it is necessary that these aids are developed in such a way that end users accept and use them; therefore, a user-centred design approach was conducted in the course of the STRANDS project to assess requirements of different stakeholders at the care site [10]. In this context, the project not only took the needs of older adults into consideration but also worked with physiotherapists to identify their needs with respect to robotic support in a caregiving environment. No specific tasks imposed from robot developers or researchers were predefined. This should enhance creativity in the consideration of potential tasks to be identified by staff in different working areas of a care facility.

The study resulted in a summary of possible tasks for robot deployment in long-term care including ideas, such as the transportation of expensable material like bandages or catheters, guiding care facility visitors or receptionist duties. As one of the more novel ideas, physiotherapists suggested that the robot could accompany the Nordic walking groups in physiotherapy [11]. In these groups, physiotherapists walk indoors with older adults with severe dementia to enhance their mobility. This scenario was chosen for implementation as robotic pacesetting for a walking group offered the opportunity to connect the project directly with daily routines in the care of older adults, while also exploring some of the wider technical challenges associated with the STRANDS project. After the implementation phase, the fully autonomous robot companion was tested in the course of the walking group sessions at the care hospital (see Appendix, section 3). For 1 month the robot accompanied the walking groups twice a week. Participating therapists and members of the research team evaluated this test phase to gain an understanding of the prevailing challenges and opportunities of this specific robot task. Prior, during and after the robot test phase, the therapists assessed their perception of the robot’s performance, looking at aspects, such as group cohesion, the amount of communication between them and the participants, as well as the mood and motivational level of participants. After the conclusion of the deployment phase the therapists were asked about their subjective experience in group interviews.

### Challenges and opportunities, therapist and developer views on robotic pacesetting

In this section, we discuss the most important issues that emerged from deployment with a focus on perceptions of staff (for more details see [11]) along with technical challenges. We also comment on some observations regarding resident interaction with the robot and explore future technical requirements.

#### Perspectives of therapists on robotic pacesetting

In general, therapists had a positive attitude towards the robot, expressing curiosity and excitement about having the robot accompany the walking groups. They appreciated the entertainment function of the robot and feedback showed that the robot had a positive influence on the participating older adults, animating them to sing or dance along, clapping their hands or whistle to the rhythm of the music. One restless older lady could use the robot as a point of orientation and thus stay closer to the group. Another participant who in the course of dementia lost the ability to speak, could connect more to the group activity due to the robot providing music. Ratings of the therapists also indicated that the robot positively influenced group coherence, motivation and the atmosphere of the group [11].

With regards to resident interaction with the robot, approximately 6 out of 10 participants tried to interact with the robot although therapists experienced that they needed guidance to use the touchscreen despite the menu being structured in a simple way. Older adults randomly pressed on the screen or pressed icons and did not release them. Another observation was that participants forgot about their plan of action during navigation through the menu due to dementia and thus needed support from the therapists. Of the participants three just looked at the robot and one older lady ignored its presence during the sessions.

Besides the evaluation of therapist and resident interactions with the robot, the deployment phase also revealed technical shortcomings. There were several instances in which technical problems impacted therapists perception of the robot. For instance, if the robot got stuck or showed other problems related to navigation, it was experienced to be more of a burden than a help.

#### Technical challenges in robotic pacesetting

To further explore technical challenges associated with the task presented before, this section focuses on robotic navigation in the caregiving environment and discusses perspectives around interaction design for long-term care settings.

##### Moving is hard… for a robot in a care setting.

The navigation abilities of the robot were most frequently reported to cause issues and as most negatively affecting the perceived usefulness of a robot in such a therapy session. From the analysis of qualitative reports obtained from therapists and augmented with information from other care staff, the following categorization of observed issues was established and underlying problems for such reported issues were identified from system logs:

1. Situations where the robot stopped moving and also after a significant waiting time (in the order of minutes) could not continue the tour: these situations were rare during the 30 days deployment of the robot and need to be attributed to the immaturity of a research prototype robot employed in the study. For example, a reason for this failure was that the on-board personal computer (PC) that had been added to increase the computational power of the original design had a power failure. Indeed, such failures have very little to do with the state of the art in research but underpin the need for scrutiny and diligence in product development to maximize availability and robustness of robots in professional applications, a requirement that is addressed by many commercial robot developers.
2. Situations in which the robot appeared to be stuck but continued after a (sometimes long) time: this technical shortcoming highlights a general challenge still evident in current state of the art robots whenever they enter a human-inhabited environment. The robot navigation software is composed of a long-established and well-matured technology^1^ so it did not fail in this case but robot navigation systems have mostly been designed to enable it to quickly move from one place to another, avoiding obstacles on the way; however, most of these obstacles are considered static, which is an assumption that clearly does not hold in a busy environment such as a care home. This problem has been addressed in a number of works, which try to overcome this problem either by modelling the dynamic flow of crowds around a robot [25] or by explicitly taking time into account when planning a route for a robot [12] or even seeking human help [14] to unblock the robot. These advances that are all aimed at making robot navigation more robust and increase the perceived safety of humans around them will eventually mature and be available off the shelf. The findings of our study only underpin the relevance of this direction of research.
3. Robot perceived as a threat: very much relating to the previous point robot navigation is considered generally safe. For a roboticist that means that the robot will not actively drive into a person and usually researchers in robotics are very much satisfied by robots efficiently navigating between places while not bumping into things and people; however, utilizing such traditional state of the art navigation approaches might well cause a robot to drive up very close to humans or plan paths that conflict with the human intention to walk along, which are situations in which the robot could be perceived as threatening.
  Navigation in humans is determined by many factors, such as conventions (e. g. passing people on the right or left depending on the cultural preference), establishing eye contact to convey intention and even assessment of others abilities. In a sense, humans negotiate non-verbally when they manage space in the proximity of other people. Enabling a robot to master this challenge even close to the way humans do it is still on-going research in the field of human-aware navigation with all its different facets [15]. Within the STRANDS project this challenge is also tackled by enabling the robot to continuously adapt to the appropriate navigation methods [5] taking the past experience of encounters with humans into account. One key challenge for this is also perception, i. e. to be able to see and recognize people in the vicinity of the robot [4], which becomes even trickier if people sit in wheelchairs or are of greatly varying posture or body height and also has to be taken into consideration. As a general policy, robots are usually designed to be very obedient to humans and if in any doubt to stop and let any humans pass first [17]. This, however, often leads to situations identified earlier where the robot does not move for a while, as it is waiting for situations that are considered to be safe to move. Consequently, it might also result in a stuttering motion, while it continuously tries to find a safe and comfortable way to move among humans, while these at the same time move as well. In a way what we observe in human-robot joint navigation is not that different to a situation we all know when humans do a little “dance” when trying to pass each other in a narrow door or corridor, while negotiating who passes on which side.

Addressing these challenges should be a key objective for upcoming research into human-aware navigation, bringing together different cues and signals that help intuitive, non-verbal negotiation of spatial movements between robots and humans.

##### Interaction design is challenging… when audiences have diverse needs.

A second major challenge besides navigation raised by the therapists was the interaction with the system itself via the touchscreen and short jingles as an auditory feedback of state changes. In the deployed therapeutic pacesetter system the interface was designed for two audiences in mind. Firstly, for the therapist with the aim to empower them to exercise full control over robot functionalities and secondly, for the participants of the therapy session to more closely involve them.
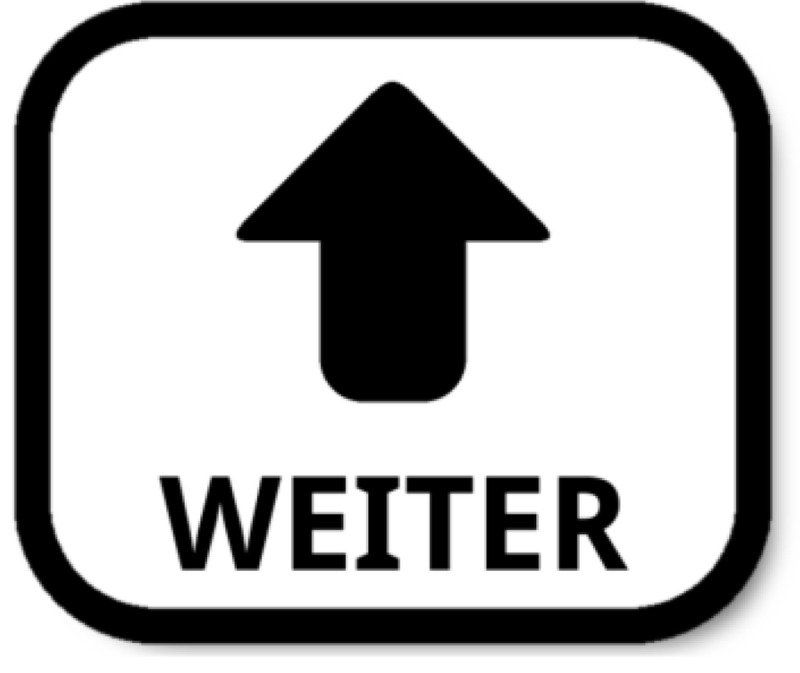


The robot switched between the two modes by recognizing the orientation of the marker shown to its left. Normally, therapists were carrying the marker upside down on a lanyard but when they wanted to stop the robot and enter the full menu that enabled more fine grained control, they could hold the marker upright to the robot to identify themselves and their intentions. This simple and intuitive authentication step prevented participants having uncontrolled and unsupervised access to all robot features. For participants, even the simplest touch interface, just showing one button “*weiter*” (German for “continue”, meant to indicate the intention to continue the tour after the robot stopped to wait for the group to catch up, see Figure on this page) turned out to be confusing as they often had no clear understanding of the semantics or had forgotten its meaning due to dementia. These occurrences add to studies that already showed that interface design for people with dementia and older adults poses particular challenges. Based on research findings, guidelines have been developed for interface design for this specific user group but observations during walking group sessions with older adults with severe dementia showed that even simple interface designs did not entirely mitigate those issues.

It is a well-known problem that speech output raises expectations in persons that the robot will also understand natural, spontaneous speech. Hence, in this setting we had explicitly disabled the speech synthesis and replaced it with audible notification (such as a jingle when the robot started to move); nonetheless, we found that many participants were anthropomorphizing the robot in terms of abilities to hold a conversation. While it is acknowledged that speech processing and understanding has made great progress, the expectations raised by the somewhat anthropomorphic design (head including eyes and general statue) of the deployed robot will still go unmet with the abilities. This ties in with general design guidelines and considerations for robots in care outlined in Wu et al. [30] and indicates that a holistic approach to interaction design is required that takes into account appearance and familiar interaction patterns.

## Lessons learned

Building on the core issues discussed in the previous section, robotic navigation in care environment and its impact on perceived usefulness of robotic aids and interaction design for diverse audiences in long-term care, this section outlines lessons learned with a focus on design, development and deployment opportunities for future projects.

1. Variation of abilities
  The care environment can be deemed to be one of the hardest for interactive mobile robots both from a navigational as well as from an interaction point of view. A robot will encounter many people with a greatly varying set of capabilities in terms of their own locomotion: people in wheelchairs cannot easily get out of the way of a robot and people using a walking aid are limited in their agility and ability to circumvent a robot. In general, as people grow older they often become more insecure about their own ability to move safely, leading to increased anxiety around a moving robot. These are challenges for robot navigation which have hardly been explicitly researched and demand much more attention of the robotics research community. The same variation of abilities is encountered when robots interact with people in a care setting. Possible interaction partners range from visitors to staff and residents, each with their own specific set of expectations, experience, and cognitive ability. The principle of designing one fits all solutions needs questioning and appropriate answers tailored for the setting, particularly when working with individuals with a range of cognitive abilities [8].
2. Low level abilities and interfaces need to fit the perceived usefulness
  From our studies we have identified that it is often the technology commonly considered to be low level in terms of robotics that poses the biggest challenges. As discussed in this article, robotic navigation and simple interaction via touchscreen or speech output are mostly considered solved in the research field; however, the specifics and variability of the care environment demand a more flexible and adaptive solution.
3. Iterative design and participatory design are essential
  The findings and considerations presented in this article stem from an in-depth analysis of data gathered in a 30-day deployment of a mobile robot platform in an real care facility. The analysis of such data is a highly interdisciplinary endeavor, requiring input from staff, sociologists and roboticists, leading to a systematic interaction analysis of exposed system behavior and internal processing models [18]. In fact, given that experience from long-term deployment of autonomous, interactive robots in care is still scarce, the community still needs to gather a lot of experience and insights into the needs and requirements, demanding iterative, participatory approaches to design and implement such systems. This calls for robotics researchers to learn more from and about needs of people being and working in care as well as the openness and enthusiasm of care institutions to learn more about and to explore the robot technology in a most integrative and interdisciplinary manner.

## Conclusion

This paper illustrates some of the challenges associated with the deployment of mobile robots in long-term care. With respect to the practical implications of our work, we believe that the following aspects need to be considered:

- Requirements analysis has to balance not only the needs of the care environment and residents but also to consider system maturity and resulting quality that can currently be delivered and be ready to adapt solutions to run reliably.
- Further efforts need to be made to explore the design of user interfaces that are suited for user groups with diverse cognitive abilities.
- Care professionals, long-term care facilities and technology developers need to collaborate on the design, development and integration of care robots in order to provide solutions that truly suit resident needs.

We hope that our paper contributes to the identification of current challenges and will help researchers and practitioners address common issues associated with robotic deployment in long-term care in the future.

## Appendix

### Infobox 1 STRANDS

STRANDS (http://strands-project.eu/) is a research project funded by the European Commission’s 7th framework programme, involving six academic partners from all over Europe and two industry partners. In its core the project aims to develop intelligent mobile robots that are able to run for months in dynamic human environments. The challenges that are particularly looked at by the STRANDS project are to provide robots with continuous learning and adaptation, which means that these robots should automatically continuously learn from their environment and the humans surrounding them and over time use the experiences they gather to become better at the services provided. Hence, the academic partners in STRANDS provide robots with the longevity and behavioral robustness necessary to make them truly useful assistants in a wide range of domains, with support in care of older adults being one of those.

The vision of the project is that such long-lived robots (robots will be running on their own for up to 120 days) will be able to learn from a wider range of experiences than has previously been possible, creating a whole new generation of autonomous systems able to extract and exploit the structure in their environments. In the care setting, this approach is expected to integrate the robot more easily into the overall workflow, adapt to user needs and to robustly cope with unexpected situations.

The robot for which STRANDS develops software is a mobile platform approximately the same size as an average human being. The robot is based on a SCITOS G5 (MetraLabs, Ilmenau, Germany) mobile robot platform equipped with various specialized sensors, enabling the robot to see and detect humans, to avoid obstacles in its way and to map out its surroundings. It is equipped with a SICK s300 laser range finder for navigation, a Kinect-like sensor mounted on a pan-tilt unit for perception of people, a touchscreen on the back and an actuated pair of eyes as a focal point for human interaction. Via speech output and touchscreen software developed within STRANDS, the robot can interact with people around it, offering information services, guidance or specialized services developed in collaboration with therapists and professionals at the care facility. The software developed by the STRANDS consortium is freely available to other researchers and interested parties, helping them to develop next generation, adaptive robots that can robustly operate for extended periods of time on their own and learn from their experience.

### Infobox 2 Robot as pacesetter

As part of the presence of the STRANDS robot at the care facility, the robot acted as a pacesetter in physiotherapy, namely the Nordic walking group. Residents with advanced dementia receive different therapeutic interventions at the care facility. One intervention is the walking group in physiotherapy with the goal to maintain the mobility of the residents, to provide them with diversion in the daily routine and to engage them in activity with other residents. During the walking group as well as in all other tasks it engaged in, the robot autonomously navigates using a navigation framework implemented by the STRANDS consortium, featuring reactive obstacle avoidance based on the sensor input and continuous path planning to move along the institution using a so-called topological map. This map redefined the coarse route for the robot and also resting areas and other actions executed during the walking group at waypoints (such as playing music in certain parts of the environment). The maximum speed of the robot during deployment was 0.55 m/s, adjusted to the speed of the quick walkers. To prevent the robot getting too far ahead of the slow walking group, certain waypoints were installed where the robot waited for the group to catch up. A specific marker detection system [13] was employed to reliably identify the therapists and allow them to take on the role of an administrator, e. g. enabling certain control functions (such as aborting the tour, rerouting and continuation) only by them. The robot autonomously identified the therapists and reacted to them accordingly. Details regarding the design and implementation can be found in Hebesberger et al. [11] but it should be noted that the system also encouraged interaction with the participants of the therapeutic session itself, by allowing them to choose music to be played during the tour or to trigger the robot to move to the next position in the tour via simple interactions on the touchscreen. Allowing the participants to engage with the robot themselves was indeed one of the explicitly desired and also regularly used features.

### Infobox 3 Deployment site

In the course of the 4‑year project STRANDS the robot platform is deployed every year at the care hospital “Haus der Barmherzigkeit” in Vienna, Austria. Founded in 1875, this care hospital specializes in the long-term care of older adults with cognitive decline, dementia as well as multimorbidities, patients with vigil coma or severe multiple sclerosis. A total of 465 employees manage and provide the long-term accomodation and nursing for 350 residents. Additionally, medical outpatient departments and departments for occupational therapy and physiotherapy are integrated into the care hospital. The robot is deployed on the ground floor of the site which includes the lobby, outpatient area and therapy sections as well as the administrative wing. In this environment the robot encounters several site-specific challenges: within the spatial deployment area much activity takes place, e. g. the transportation of residents either by foot, in wheelchairs or beds from units to the outpatient or therapy areas and the coming and going of employees and visitors. This constitutes a very busy environment through which the robot has to navigate; furthermore, the robot tasks have to meet the interests and abilities of various groups of potential users, such as doctors, therapists, nursing staff, visitors and residents with dementia. Thus, interesting contents or tasks have to be integrated into easily structured menus or routines.

## Footnotes

1 Most of the robot software is built using the robot operating system (ROS http://www.ros.org/) that also contains a navigation subsystem, which can be considered the most commonly used within the robotics research community.
